# The South American Society of Cardiology (SSC) and the Latin American Association of Cardiac and Endovascular Surgery (LACES) Statement on the 2021 ACC/AHA/SCAI Guidelines for Coronary Artery Revascularization

**DOI:** 10.21470/1678-9741-2023-0119

**Published:** 2023-08-07

**Authors:** Gerardo Soca, Alejandro Martínez, Walter J. Gomes, Jose Guillermo Solano, Rui Almeida, Maria Alejandra Ibañez, Mateo Marin-Cuartas, Graciela Hurtado, Victor Dayan

**Affiliations:** 1 Centro Cardiovascular Universitario, Universidad de la Republica del Uruguay, Montevideo, Uruguay; 2 Division of Cardiovascular Diseases, Pontifícia Universidad Católica de Chile, Santiago, Chile; 3 Cardiovascular Surgery Discipline, Hospital São Paulo, Escola Paulista de Medicina, Universidade Federal de São Paulo, São Paulo, São Paulo, Brazil; 4 Centro Médico Medicina Externa S.A. (MEDEX), San Isidro, Peru; 5 Faculdade de Medicina, Centro Universitário Fundação Assis Gurgacz, Cascavel, Paraná, Brazil; 6 Cardioayuda Ltda. y Sociedad Colombiana de Cardiología y Cirugía Cardiovascular, Bogotá, Colombia; 7 University Clinic for Cardiac Surgery, Leipzig Heart Center, Leipzig, Germany; 8 Seguro Integral de Salud (SINEC) – Seguridad Social y Unidad de Diagnóstico Médico (UDIME), Santa Cruz de la Sierra, Bolivia

The Latin American Association of Cardiac and Endovascular Surgery (LACES), partnering with the South American Society of Cardiology (SSC), analyzing the 2021 American College of Cardiology (ACC)/ American Heart Association (AHA)/Society for Cardiovascular Angiography and Interventions (SCAI) Guidelines for Coronary Artery Revascularization^[1]^, has reached the conclusion that there is not enough evidence to endorse the recommendations included in its Chapter 7, focusing revascularization in stable ischemic heart disease. This decision is consistent with statements from other prestigious scientific societies: American Association of Thoracic Surgery^[2]^, Society of Thoracic Surgery, Latin-American Association of Cardiac and Endovascular Surgery, European Association of Cardiothoracic Surgery, Japanese Society of Cardiovascular Surgery, and British Society of Cardiovascular Surgery; and it is mainly based on the downgrade of the class of recommendation (COR), from I to IIb, for coronary artery bypass grafting (CABG) as a treatment to improve survival in patients with stable three-vessel coronary artery disease (CAD) with preserved left ventricular function and no left main CAD. This statement is of paramount importance since this change in the level of recommendation dismissing the survival benefit of CABG in this scenario may result in compromise of medical coverage for millions of patients worldwide and particularly in Latin America whom otherwise could benefit from this intervention.

## RATIONALE

The primary rationale for this change was twofold: the trials supporting the former COR for CABG *vs. *optimal medical treatment (OMT) were completed > 20 to 40 years ago and no longer embodied modern OMT; and also by inference from preliminary analysis of the CABG subgroup of the International Study of Comparative Health Effectiveness With Medical and Invasive Approaches (ISCHEMIA) trial^[3]^.

More recent analysis contradicts the first argument. The Medicine, Angioplasty, or Surgery Study (MASS-II) trial, the sole trial ever to compare CABG, angioplasty (percutaneous coronary intervention [PCI]), and OMT in patients with multivessel CAD, stable angina, and preserved ventricular function, reported in 2010 the 10-year follow-up, reinforcing the results of earlier studies while adding new insights^[4]^. In the MASS-II trial, all patients were placed on an optimal medical regimen at baseline until the end of follow-up, consisting of aspirin, β-blockers, angiotensin-converting enzyme inhibitors, calcium channel blockers, nitrates, and lipid-lowering agents, along with low-fat diet, on an individual basis. All medications were dispensed free of charge to all patients throughout the 10-year follow-up to ensure protocol adherence. Proximal left anterior descending (LAD) CAD was present in 89% of patients in the OMT group and 93% of the CABG patients. Although the study design was heavily underpowered for assessing individual components of the composite endpoint, a significantly lower incidence of non-fatal myocardial infarction (Ml) with CABG *vs. *OMT was seen at 10 years (20.7 *vs. *10.3, *P*=0.010) but not at five years (*P*=0.785). Cardiac death was not significantly different at five years but higher with OMT *vs. *CABG (20.7% *vs. *10.8%, *P*=0.019) at ten years. Overall mortality was reduced with CABG *vs. *OMT (25.1% *vs. *31.0%, *P*=0.089), although not reaching statistical significance. The pairwise comparison analysis showed a significant 2.02- and 2.77-fold increased risk of cardiac death and subsequent Ml with OMT *vs.* CABG, respectively, demonstrating the progressively better long-term prognosis of surgical patients. The results of the MASS-II trial are comparable to the findings of the Surgical Treatment for Ischemic Heart Failure (or STICH)^[5]^, Future Revascularization Evaluation in patients with Diabetes mellitus: Optimal management of Multivessel disease (or FREEDOM)^[6]^, and SYNergy between percutaneous coronary intervention with TAXus and cardiac surgery (or SYNTAX) trials^[7]^, where additional and robust benefits from CABG were steadily growing at longer-term follow-up beyond the five-year scrutiny.

Additional evidence used to support the downgrade in recommendation stemmed from meta-analyses in which revascularization is compared to medical treatment. In these studies, CABG and PCI are considered equivalent revascularization strategies, which is a misconception given the different impacts of both procedures in reducing spontaneous Ml and long-term mortality. Furthermore, the number of patients who underwent CABG in these pooled analyses is significantly lower than that of patients who underwent PCI^[8]^.

Next, the ISCHEMIA trial was neither designed nor statistically powered to compare CABG with OMT. In particular, the CABG group was not powered for detecting potential mortality differences, and the follow-up is restricted to a median of 3.2 years. The primary outcome was the composite of death from cardiovascular causes, Ml, or hospitalization for unstable angina, heart failure, or resuscitated cardiac arrest. The method of revascularization in the invasive group — percutaneous or surgical — was not randomized in the ISCHEMIA trial. CABG represented only 25.8% of all revascularization procedures and was performed in 22.3%o of patients in the initial invasive strategy, also, it was indicated only when PCI was not the best option due to the extent and severity of CAD. Therefore, comparing CABG *vs. *OMT from ISCHEMIA is inappropriate due to a strong selection bias. The patients enrolled in the ISCHEMIA trial were not representative of patients with multivessel CAD and typically referred to guideline-based CABG; fewer than half had proximal stenosis of the LAD coronary artery. Therefore, the overall analysis of the ISCHEMIA trial cannot be applied to this subset of more complex patients who underwent CABG. Matching CABG and OMT patients by anatomy is impracticable, and this comparison would not provide any high-quality causal inference, as it would be underpowered, not pre-specified, or randomized. It is illogical that such implied effects were used to guide the revision recommendations; therefore, the ISCHEMIA trial did not provide any new or consistent evidence to invalidate the previous Class 1 level of evidence A for CABG in multivessel coronary disease. Of the 5,179 patients of the ISCHEMIA trial, only 2% had Duke 6 and underwent CABG. Data derived from the under-representation of the population of interest should not be considered evidence for the current recommendation^[9]^.

Further analysis of the ISCHEMIA trial revealed that more severe CAD was associated with an increased risk of Ml (both spontaneous and periprocedural Ml subtypes), higher risk of cardiovascular death, the trial primary composite endpoint, and cardiovascular death or Ml. The outcomes of the typical multivessel CABG patient contemporarily referred by heart teams, *i.e., *three-vessel severe stenosis (≥ 70%o) or two-vessel severe stenosis with proximal LAD (defined by the authors as modified Duke Prognostic Index score of 6), showed a significant reduction in the four-year rate of cardiovascular death or Ml in the invasive strategy group (difference, 6.3% [95% confidence interval 0.2%–12.4%])^[6,7]^. The ISCHEMIA trial included less than one-third of the patients in this category. Additionally, the anatomic completeness of revascularization in the CABG group was 34%, and the functional was 48.5%), clearly below the expected rate once compared to current surgical practice^[9,10]^.

We acknowledge that OMT has made a giant leap forward since the earlier RCT trials, but so does CABG with technical refinements and outstanding contemporary outcomes. But we believe previous recommendations should prevail until new evidence emerges from studies designed to evaluate the benefit of CABG *vs. *medical treatment in patients with severe three-vessel disease and preserved LVEF^[11]^.

In summary, LACES and SSC do not agree on critical aspects of the 2021 ACC/AHA/SCAI guideline on coronary artery revascularization. As scientific societies, we do not endorse the recommendations of Chapter 7. The downgrade of the CABG recommendation for patients with the three-vessel disease is unjustified and stems from gross misconceptions while ignoring previous solid evidence. Therefore, until new evidence addressing CABG *vs. *medical treatment in patients with the three-vessel disease is available, it is prudent to retain the Class I recommendation for CABG to improve long-term survival in the subset of patients with three-vessel disease, stable ischemic disease, and preserved LVEF.
